# Automatic Count of Bites and Chews From Videos of Eating Episodes

**DOI:** 10.1109/access.2020.2998716

**Published:** 2020-06-01

**Authors:** DELWAR HOSSAIN, TONMOY GHOSH, EDWARD SAZONOV

**Affiliations:** Electrical and Computer Engineering Department, The University of Alabama, Tuscaloosa, AL 35487, USA

**Keywords:** Meal microstructure, computer vision, deep learning, image classification, optical flow, bite count, chew count

## Abstract

Methods for measuring of eating behavior (known as meal microstructure) often rely on manual annotation of bites, chews, and swallows on meal videos or wearable sensor signals. The manual annotation may be time consuming and erroneous, while wearable sensors may not capture every aspect of eating (e.g. chews only). The aim of this study is to develop a method to detect and count bites and chews automatically from meal videos. The method was developed on a dataset of 28 volunteers consuming unrestricted meals in the laboratory under video observation. First, the faces in the video (regions of interest, ROI) were detected using Faster R-CNN. Second, a pre-trained AlexNet was trained on the detected faces to classify images as a bite/no bite image. Third, the affine optical flow was applied in consecutively detected faces to find the rotational movement of the pixels in the ROIs. The number of chews in a meal video was counted by converting the 2-D images to a 1-D optical flow parameter and finding peaks. The developed bite and chew count algorithm was applied to 84 meal videos collected from 28 volunteers. A mean accuracy (±STD) of 85.4% (±6.3%) with respect to manual annotation was obtained for the number of bites and 88.9% (±7.4%) for the number of chews. The proposed method for an automatic bite and chew counting shows promising results that can be used as an alternative solution to manual annotation.

## I. INTRODUCTION

Study of eating behavior of individuals is important for researchers to understand the eating patterns of people suffering from obesity and eating disorders. In order to understand the complex process of dietary habits, it is necessary to track the dynamic process of eating episodes which are known as meal microstructure [1]–[3]. Meal microstructure combines factors of food intake behavior such as total eating episode duration (start of the food intake to the end including pauses), true ingestion duration (time spent in chewing), eating event number (a bite which is potentially followed by a segment of chewing and swallowing), ingestion rate, frequency and efficiency of chewing, and size of bite [4]. As the meal microstructure is used to characterize the eating behavior of an individual, studies of meal microstructure may provide new insights and findings in the treatment of obesity. In [5], the authors claimed that a reduction in bite-rate results in the lower intake. Other studies have shown that there is a clear relationship between food intake rate and energy intake [6]–[9]. In [9], the authors suggested that to fight against obesity, interventions to improve chewing activity could be a necessary tool. In [6], the authors claimed that eating rate feedback can be helpful to aid in intervention in eating disorder treatment. Health researchers are applying the number of chews and the rate of chewing as variables in their models for estimation of ingested mass and energy intake. Counts of chews and swallows were used to develop the energy intake estimation model (CCS) in [10]. In [11] authors concluded that for solid food, recordings of chewing sound can be used to predict bite weight.

Recently, many health researchers are exploring several wearable devices to monitor food intake behavior and record meal microstructure in eating episodes [12]. Different sensor modalities such as acoustic sensors [11], [13], accelerometers [14], [15], surveillance video cameras [16], smartphone cameras [17], piezoelectric sensors [18], [19], motion sensors [5], hand gesture sensors [20] are being explored in development of these wearable devices. To train and validate sensor-based models, most of the studies of food intake monitoring systems [6]–[8], [10], [19], [21]–[24] apply manual annotation of chew count by either participants or the investigators. Although video observation and manual annotation is an accepted gold standard for these studies, the manual chew count can be inaccurate, time-consuming and a burden to the investigators when the data size is large. In [25], the authors used both manual annotation of videos and sensor-driven features to quantify energy intake. They mentioned that manual analysis is not realistic on a large scale. Hence an algorithm to make the annotation automatic in feeding studies is necessary.

Little work has been done so far to make the annotation from eating episode videos automatic. In [26], the authors used face detection and a variable-intensity template modeling to track changes in luminance of the left and right cheeks and chin areas during chewing. They achieved an accuracy of 83% on a small dataset (480 chews) and detected faces only from the front on a limited number of foods, whereas chewing depends upon food type. In [16], the authors used visual quasi-periodicity in chewing to detect chewing events from videos using support vector machines. This model worked only to detect chewing events (classification) while not counting the number of chews. Recently, in [27] authors developed a deep network using features from both face and body to detect bite instances and count the number of bites, but did not count the number of chews in the eating episodes. A novel deep learning based algorithm named “Rapid Automatic Bite Detection” (RABid) [28] was developed by the authors that extracts and processes skeletal features from videos of eating episodes to measure meal duration and bites using long short-term memory (LSTM) network. None of the above-mentioned methods can be used as a fully automatic alternative to human meal-video annotations for the experimental analysis of human eating behavior, as they don’t calculate bites and chews of the meal together.

In this paper, we propose a novel contactless bite and chew counting method from video based on object detection, image classification, and optical flow. The proposed method combines automatic face detection, and bite and chew counting to provide a fully automatic approach for annotation of food consumption documented on video.

## II. METHODS AND MATERIALS

### A. DATA COLLECTION PROTOCOL

**FIGURE 1.**
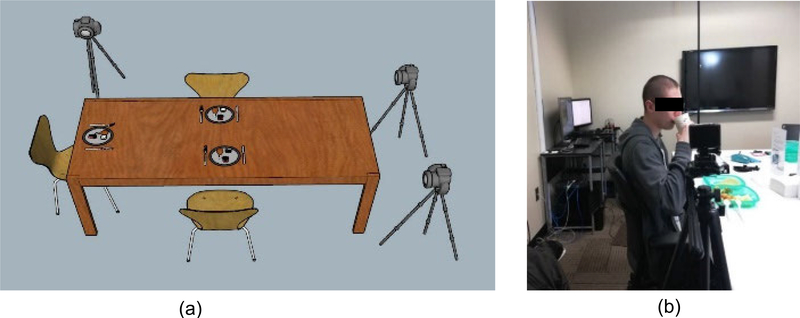
Camera and data collection set up (a) Data collection setup (b) Real participant.

**FIGURE 2.**
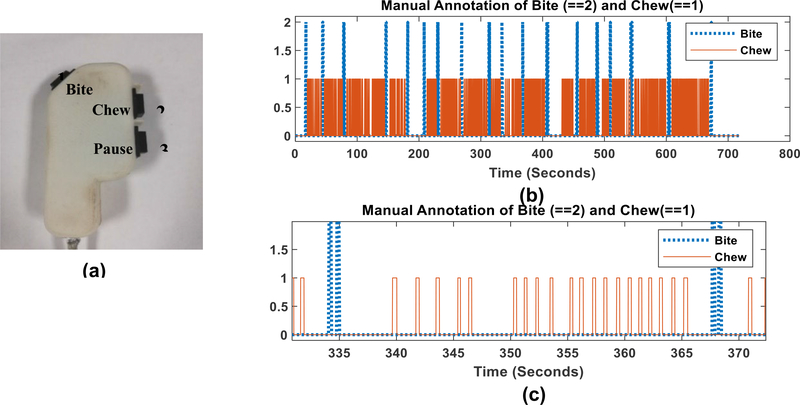
Manual annotation of bite and chew from video (a) The three button system for annotation the video (b) Annotated bite and chews in a meal video (c) Annotated bite and chew of a chewing sequence.

A total of 28 volunteers were recruited (17 males and 11 females, average age 29.03 ± 12.20 years, range 19–41 years, average body mass index (BMI) 27.87 ± 5.51 kg/m2, range 20.5 to 41.7). Participants were asked to consume three free meals (breakfast, lunch, and dinner) in the laboratory where the eating was recorded on video. Participants were taken to on-campus food courts at the University of Alabama to self-select their meals. There was no restriction on the choice of food items. Several participants (up to 3) were invited to the laboratory at the same time to simulate social eating. This study was approved by The University of Alabama’s Institutional Review Board. Recruited participants did not show any medical conditions which would hinder their normal eating or chewing. All participants read and signed an Informed Consent before participating in the study. The meal episodes were recorded using an SJCAM SJ4000 Action Camera (Black).This camera takes 1080p video at 30 frames per second. Fig. 1 shows the data collection setup in the laboratory. The cameras were positioned at 3 feet away from participants. The cameras were positioned to take video in the profile view to make the tracking of the jaw movement easier for manual annotation. The subjects were instructed to eat in a natural way which can include talking and gestures with a self-determined pace. The video dataset contained a total 19 Hours and 26 Minutes videos of 84 meals from 28 different subjects. Because the highest frequency of chewing is about 2Hz, the recorded videos were down-sampled into 6fps image frames. The dataset contained 419737 image frames, 2101 bites and 45581 chews that were counted using manual annotation of the video. The manual annotation process was performed using a 3-button system and custom made LabView software as described in [29]. The three-button system shown in Fig. 2(a) contains dedicated buttons to count number of chews and bites. The meal video was played in a monitor with 5x slower speed and bite and chewing events were marked by pressing the button. One complete meal video annotation is shown in Fig. 2(b) and a short sequence of a bite followed by chewing is shown in Fig. 2(c). All the computation and processing was performed offline in MATLAB 2018b (Mathworks Inc., Natick, MA, USA).

### B. FACE DETECTION TO EXTRACT ROI

**FIGURE 3.**
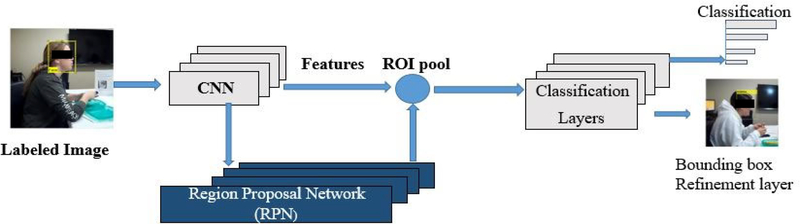
Architecture of faster R-CNN object detector.

**FIGURE 4.**
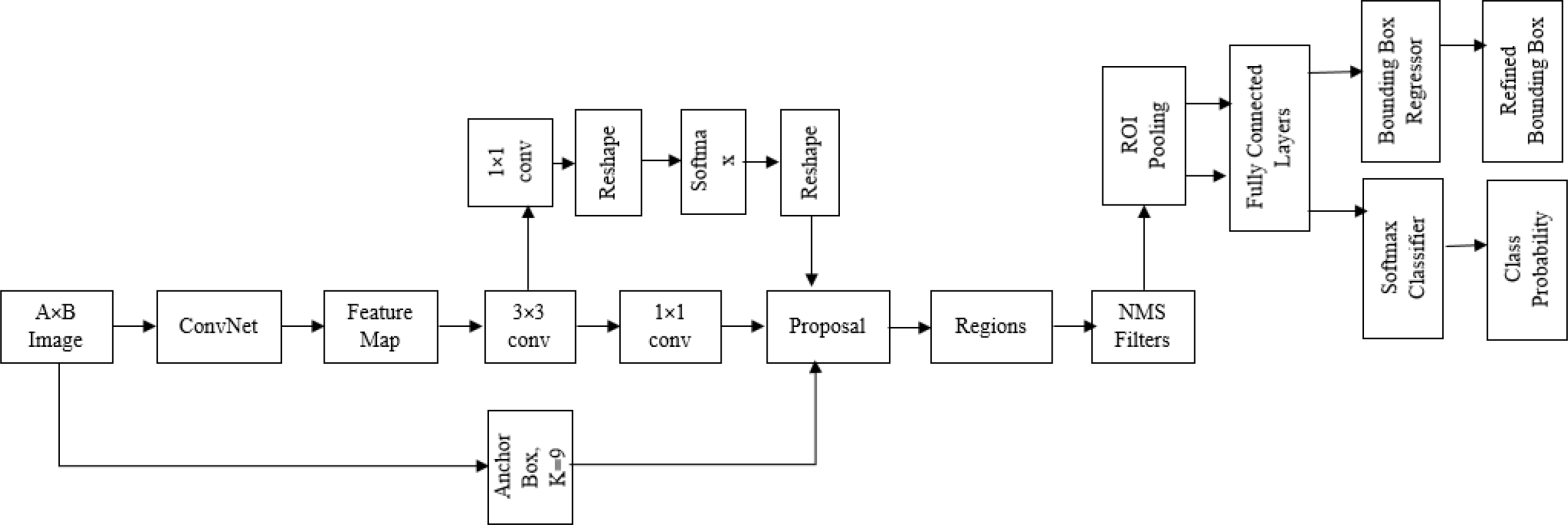
Structure and layers of face detector.

In the first step of processing, we converted the meal videos into 6 fps image frames. The we detected the region of interest (ROI) from the converted image frames of the videos. As the goal of this project is to automatically count bites and chews, only the face of the participant is the ROI. To detect the face from images, deep-learning based object detection algorithm was used. In recent years, deep Convolutional Neural Networks (CNNs) became very popular in computer vision applications. For object detection, region-based CNN detection methods are becoming the primary choice for researchers. In order to bring more speed and accuracy in object detection, three generations of region-based CNN detection models, from the R-CNN [30], to the Fast R-CNN [31], and finally the Faster R-CNN [32], have been proposed within the space of few years. In this project, Faster R-CNN was used to detect faces in image frames. Fig. 3 shows the architecture of the Faster-RCNN object detection algorithm. Typically, any RCNN based object detection [33] algorithm consists of two stages: 1) a Region Proposal Network (RPN) consists of a fully convolutional network which is responsible for generation of specific region proposals for each image, and 2) a deep convolution network which is used for pooling of region proposals and object detection. As most of the computation of the RPN stage and object detection network stage is shared, a single deep network (e.g. CNN) can be trained to serve in both stages and produce high-quality proposals, pooling of the proposals, and object detection. Fig. 4 represents the structure and layers of the trained object detection network. We used the transfer learning approach with a ResNet-50 network trained on ImageNet dataset to classify images into 1000 catagories which consists 50 convolution layers. To produce a fixed-size feature vector, the final layer of ResNet-50 was followed by a 3 × 3 convolutional layer [33]. The region proposal network produced 9 proposals with three different aspect ratios (1:1,1:2, 2:1) and three different scales (32, 64 and 128 pixels), at each place which was parameterized relative to an anchor box called reference box. For classification and regression, the output was then passed to a 1 × 1 class score layer and regression layer. The regression layer provided a 4 × 9-dimensional output which included coordinates of the corner for all anchors of the bounding box. The class score layer provided a 2 × 9 dimensional output which included a score for both the object and background. We used ResNet-50 trained on ImageNet dataset with 1000 classes. So the output of the CNN is 50. But the last fully connected layer, the softmax layer and classification layer were replaced by a fully connected layer, a softmax layer and a classification layer with two classes (Face and background).To predict the class and bounding box refinement, the region proposals generated by RPN and the convolutional features, both were passed into the ROI (region of interest) pooling layer. Fig. 4 represents the structure and layers of proposed face detector.

**FIGURE 5.**
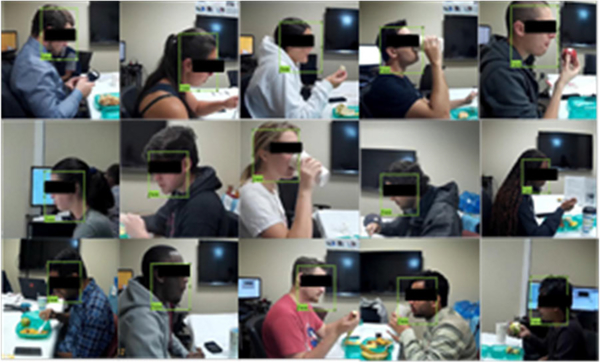
Labeled training Image for face detector.

The meal videos were recorded with a resolution of 1920 × 1080. In order to remove the surroundings, the image frames were cropped with fixed coordinates to the size of 840 × 760. To train the object detector, 300 image frames from recorded meal videos of each participant were randomly selected, which provided a total of 8400 training images. In order to label the training image, ImageLabeler API [34] was used on the image set and a human rater manually marked the boundary (the bounding box) of the face as the object to be identified. Fig. 5 represents the labeled training images. In the training stage, 4-fold cross-validation method was implemented. The images of 21 subjects (6300 images) were used for training at once and the rest of the 7 subject’s images (2100 images) were used in validation. To increase the size of the dataset, image augmentation and resizing procedures were implemented on the whole training image set. The image set was augmented by a) Y-reflection mirror, b) 2-D Gaussian smoothing (blurring), c) brightness adjustment, d) orientation change to portrait mode, and e) crop and rotation by clockwise 10 degrees and anticlockwise 10 degrees. The post augmentation image set contained 42700 training images at each fold. Learning rate, batch size and the number of epochs were chosen as 0.001, 1 and 4 respectively which were empirically selected from grid search procedures. As we used ResNet-50 network trained on ImageNet dataset, to reduce the chance of overfitting the number of epochs were set as low as 4 in fine tuning.This training was performed on a MATLAB 2018b environment installed on a Windows 10 computer equipped with an Intel Core i7 9th Generation CPU with 16GB DDR4 RAM, and an NVIDIA GeForce GTX 1070 GPU with 8GB memory.

### C. IMAGE CLASSIFICATION TO COUNT BITES

**FIGURE 6.**
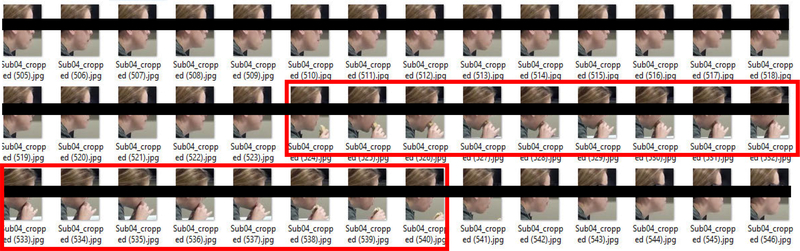
Cropped faces of an eating episode to detect bite.

**FIGURE 7.**
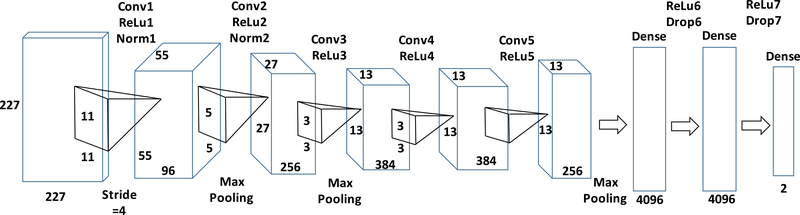
Structure and layers of image classifier to classify bite.

A bite can be defined as the process of taking the liquid, semi-solid or solid food by the person using straw, glass, bottle, spoon, fork or hand [35], [36]. We used detected faces from image frames to count the number of bites in an eating episode by detecting faces with straw/glass/bottle/hand/spoon/fork and food in the field of view (defined as ‘bite’ image). The images containing only faces were considered as ‘non-bite’ images. Fig. 6, shows a sequence of cropped faces of an eating episode with the ‘bite’ images highlighted by the box. We used a pre-trained AlexNet [37] to train a binary image classifier to classify cropped faces as ‘bite’ image or ‘non-bite’ image. The Faster RCNN network used in face detection, consists 50 convolutional layer for classification which could be used in bite vs no bite classification. Instead we used a separate pre-trained network named AlexNet, which consists 5 convolutional layer to reduce computational cost, training time and overfitting. The structure and layers of the proposed classifier from pre-trained AlexNet are presented in Fig. 7. The structure of AlexNet comprises of 5 convolution layers followed by 5 rectified linear activation units (ReLU). The first two Convolutional layers are followed by overlapping max pooling. The third, fourth and fifth convolutional layers are connected directly. The fifth convolutional layer is followed by an overlapping max pooling which output is feed into a series of two fully connected layers. The last fully connected layer goes into a softmax classification layer. Batch normalization was performed between input layer and first two convolutional layers. For bite image classification, the AlexNet network trained on ImageNet were retrained for binary classification. As the cropped faces were classified into the bite or non-bite image, thus, the fully connected layer had two outputs. The input layer of the network was fed by a 227 × 227×3 color image. The first convolution layer consisted of 96 kernels of size 11 × 11×3 with a stride of 4 pixels which passed the input to the second convolution layer through normalization and pooling. The second, third, fourth and fifth convolution layer had 256 kernels of size 5 × 5 × 48, 384 kernels of size 3 × 3 × 256, 384 kernels of size 3 × 3 × 192 and 256 kernels of size 3 × 3 × 256 respectively. The fully connected layers had 4096 neurons each. The images with cropped faces resized to 227 × 227 × 3 were used to retrain the last three layer of the described network. The training dataset contains 2800 bite images and 5600 no bite images. During training 4 fold cross-validation and image augmentation schemes were applied as described in Face Detection and Extract ROI section. The two dropout layers drop neurons that have a probability of 0.5 or less. Learning rate, batch size and the number of epochs hyperparameters were chosen as 0.001, 10 and 6 respectively which were empirically selected from grid search procedures. This training was performed on a MATLAB 2018b environment installed on a Windows 10 computer equipped with an Intel Core i7 9th Generation CPU with 16GB DDR4 RAM, and an NVIDIA GeForce GTX 1070 GPU with 8GB memory.

### D. OPTICAL FLOW TO COUNT NUMBER OF CHEWS

In order to track the movement of the jaw while chewing, we used optical flow, a computer vision approach that estimates the apparent motion of surfaces, edges, and objects between images [38]–[41]. The motion between two image frames which are taken at times t and t+∇*t* can be interpreted by calculating the motion at every pixel location. The images are represented as 3-D vector fields, *I*(*x, y, t*) where (*x, y*) and *t* denotes the spatial coordinates and time, respectively. For a specific time instance pair of images, the spatial motion, ***u*** = (*u*_*x*_*, u*_*y*_), is estimated for every pixel. The basic assumption behind the idea of optical flow is the brightness constancy assumption, which states that the brightness (intensity) of a pixel remains constant during its motion, and for a specific pixel, it is expressed as

$$I_{x}u_{x}+I_{y}u_{y}+I_{t}=0$$

where *I*_***x***_, *I*_***y***_, and *I*_***t***_ denote the partial derivatives of the image function in the x-axis, y-axis, and time, respectively. As the equation has two unknowns *(u*_***x***_, *u*_***y***_), it does not have any unique solution which is the mathematical consequence of aperture problem, which is defined as the lack of information in a small area to determine motion. To solve the problem, additional constrains need to be introduced. There are several approaches which bring additional constraints to find the optical flow, such as Lucas-Kanade method [38], Horn-Schunk method [40], Buxton-Buxton [42], and Black-Jepson method [41]. The constraints imposed by Lucas-Kanade method suggest that the displacement of the contents of two nearby image frames is quite small and can be considered constant within a neighborhood of the point p under consideration. Hence the local image flow (velocity) vector ((*u*_*x*_, *u*_*y*_) must satisfy for all pixels within a window centered at p. If we use a 5 × 5 window, that gives us 25 equations per pixel.

$$\;Ad=b,\;where\;A_{25\times 2}=\left[\begin{array}{ll} I_{x}(p_{1}) & I_{y}(p_{1})\\ I_{x}(p_{1}) & I_{y}(p_{1})\\ \cdot & \cdot \\ \cdot & \cdot \\ I_{x}(p_{25}) & I_{y}(p_{25}) \end{array}\right]\;d_{2\times 2}=\left[\begin{array}{c} u\\ v \end{array}\right]\;and,\;b_{25\times 1}=-\left[\begin{array}{l} I_{t}(p_{1})\\ I_{t}(p_{2})\\ \cdot \\ \cdot \\ I_{t}(p_{25}) \end{array}\right]$$

Now the system has way more equations than unknowns and thus is commonly over-determined. The Lucas-Kanade method obtains a solution by using least squares principle *d = b*; minimize ∥*ad* – *b*∥^2^

The minimum least squares solution: *(A*^*T*^*A)d = A*^*T*^*b* which can be written as following

$$\left[\begin{array}{c} u\\ v \end{array}\right]=-{\left[\begin{array}{cc} \sum I_{x}I_{x} & \sum I_{x}I_{y}\\ \sum I_{x}I_{y} & \sum I_{y}I_{y} \end{array}\right]}^{-1}\left[\begin{array}{c} \sum I_{x}I_{t}\\ \sum I_{y}I_{t} \end{array}\right]$$

**FIGURE 8.**
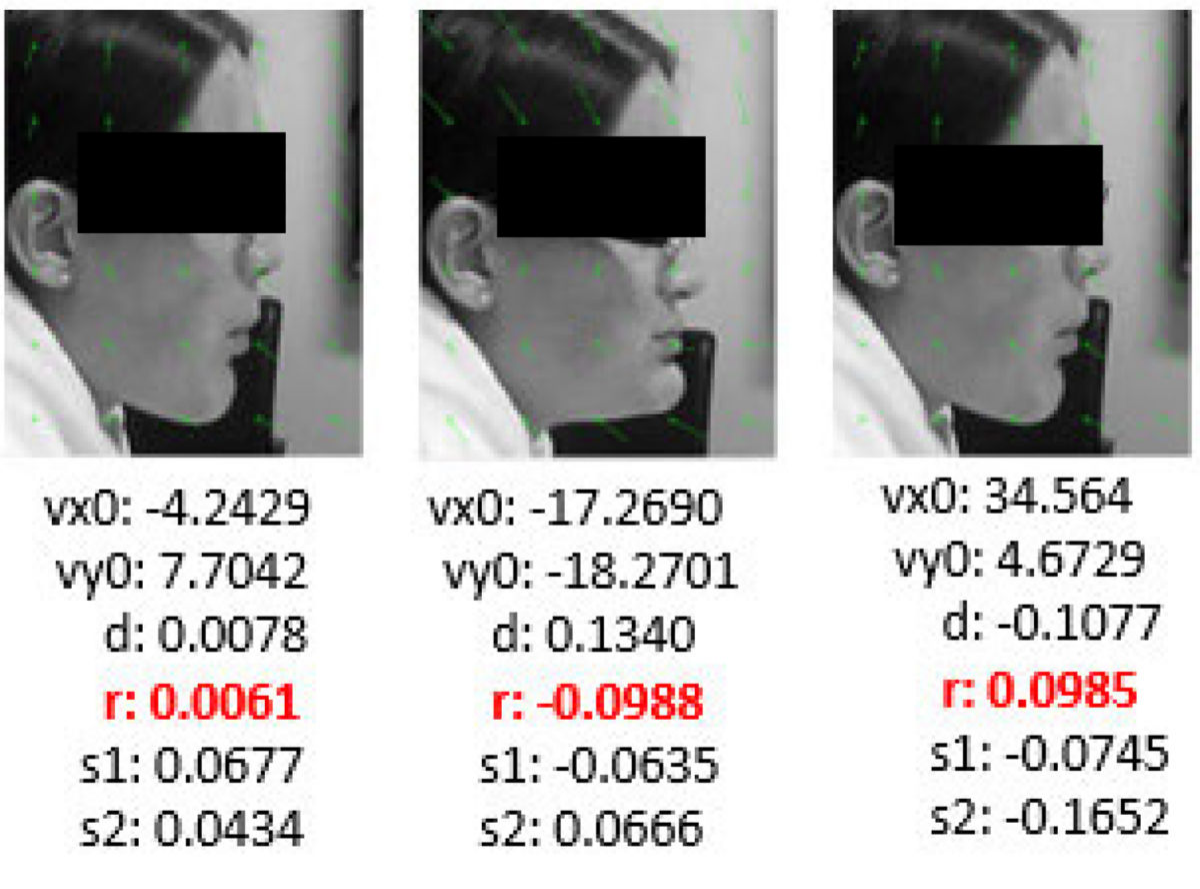
Affine optical flow to find chewing. First and last image show the jaw opening and the middle image shows the jaw closing movements during chewing.

In order to detect chewing, we used affine (or first-order) optical flow model which has 6 parameters: two to describe the translational movement in x (*vxo* ) and y-direction *(vy0);* one to describe rotation (*r*); two to describe angular sheer movement *(s1 and s2);* and one for directional movement (d). A least-squares fit of the parameters, which is an extension of the Lucas-Kandle method [43], was implemented to estimate the spatial and temporal grey-level gradients. In order to apply optical flow, each image frame was passed through the face detector. The detected face was cropped, converted to a grayscale image and resized to 150 × 120 pixels. Fig. 8 shows the effect of optical flow in consecutive images while chewing. Chewing is associated with opening and closing the jaw muscles during mastication [44]. The values of the rotational parameter (*r*) of optical flow are the highest during jaw opening and the lowest during jaw closing. This phenomenon suggests that the rotational parameter of optical flow can be used to explain chewing. The translational movement in the y-direction (*vy0*) also shows similar characteristics. But as the grinding movement of the jaw during chewing can be best described by the rotational movement, we used the rotational parameter.

### E. BITE AND CHEW COUNTING

The pipeline of developed automatic chew and bite counting from meal episode video is represented in Fig. 11. The algorithm for counting the number of bites and chews from videos of eating episodes is presented in Fig. 12. First, the recorded 30 fps meal video was converted to 6fps image frames. Each frame is then passed through the face detector. If no face was detected or the confidence level of detection was below 0.8, then the frame was ignored and a new frame was taken as input. The detected faces were cropped, resized and then passed through two processing steps 1) bite/non- bite image classification, and 2) calculation of affine optical flow parameter to be used in chew counting.

**FIGURE 9.**
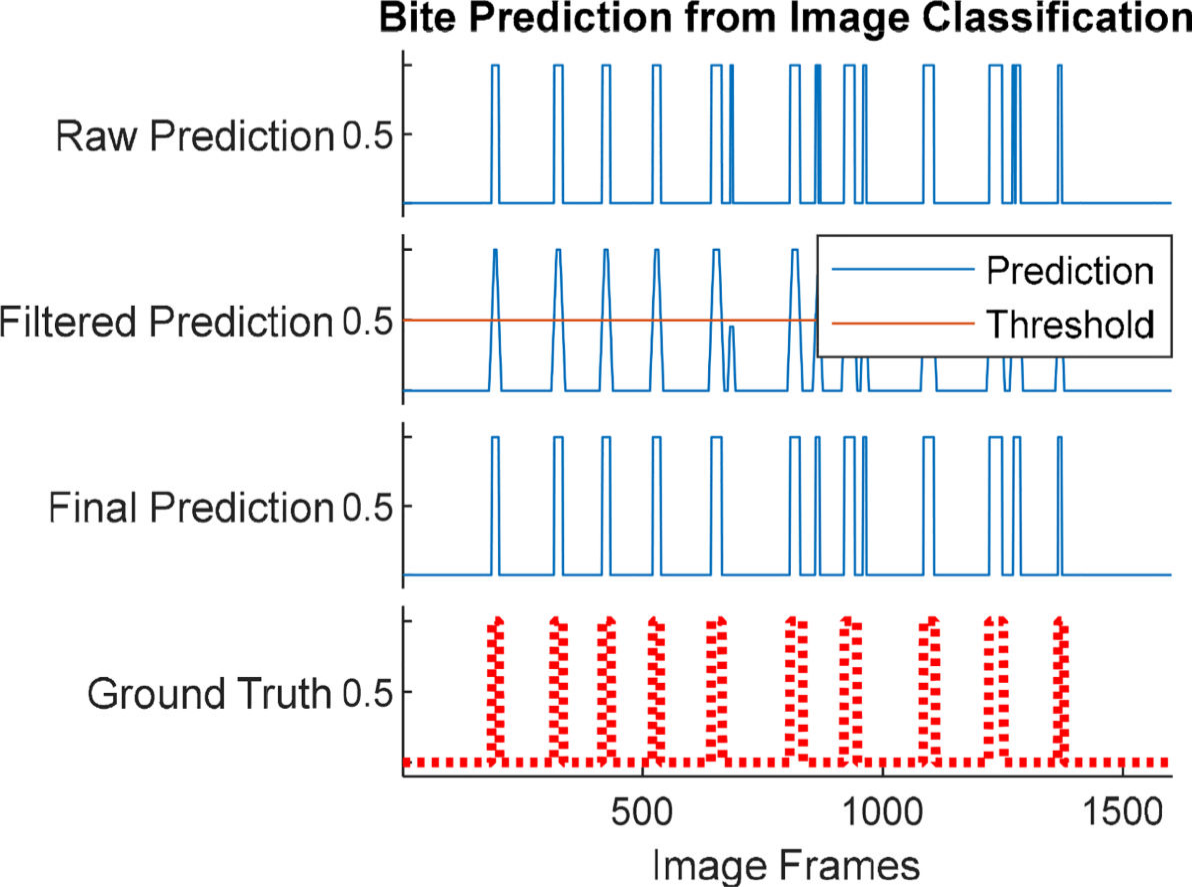
Prediction of bite count from image classification. (Top figure represents raw prediction, middle two figures represents filtering and thresholding to remove false detection and the bottom figure represents the ground truth obtained from manual annotation).

The counting of chews took several considerations into account. A typical sequence of solid food consumption consists of a bite followed by a chewing sequence. Consumption of fluids consists of a bite (placing liquid in mouth) that is not followed by chewing. Also, gestures such as the use of napkin to wipe mouth may be detected as a bite but followed by no chewing. Fig. 9 shows the bite recognition of an eating episode. The top graph shows the raw prediction from cropped faces (bite images labeled as ‘1’ and non-bite images labeled as ‘0’). In order to remove false detection, the classification labels were smoothed by a moving average filter with a span of 10 images. The smoothed classification results were binarized by a threshold with a value of 0.5. This false detection may cause over-prediction in chew counting. Thus, chewing segments were identified by calculating the short-time energy of the signal between consecutive bites and estimating a dynamic threshold (*T*) as described in [22]. If the short-time energy of the optical flow signal between consecutive bites was below the threshold, then that segment was not used in counting the chews. Another consideration is that food consumption is not a continuous process. People often take rest after taking a bite and chewing the food. The movement of the head during these rest periods can also produce false chew count.

From the experimental data we determined that the longest observed chewing sequence was 52 seconds in duration. Thus, at the most 52 seconds of optical flow signal was used in the calculation of chew counting.

**FIGURE 10.**
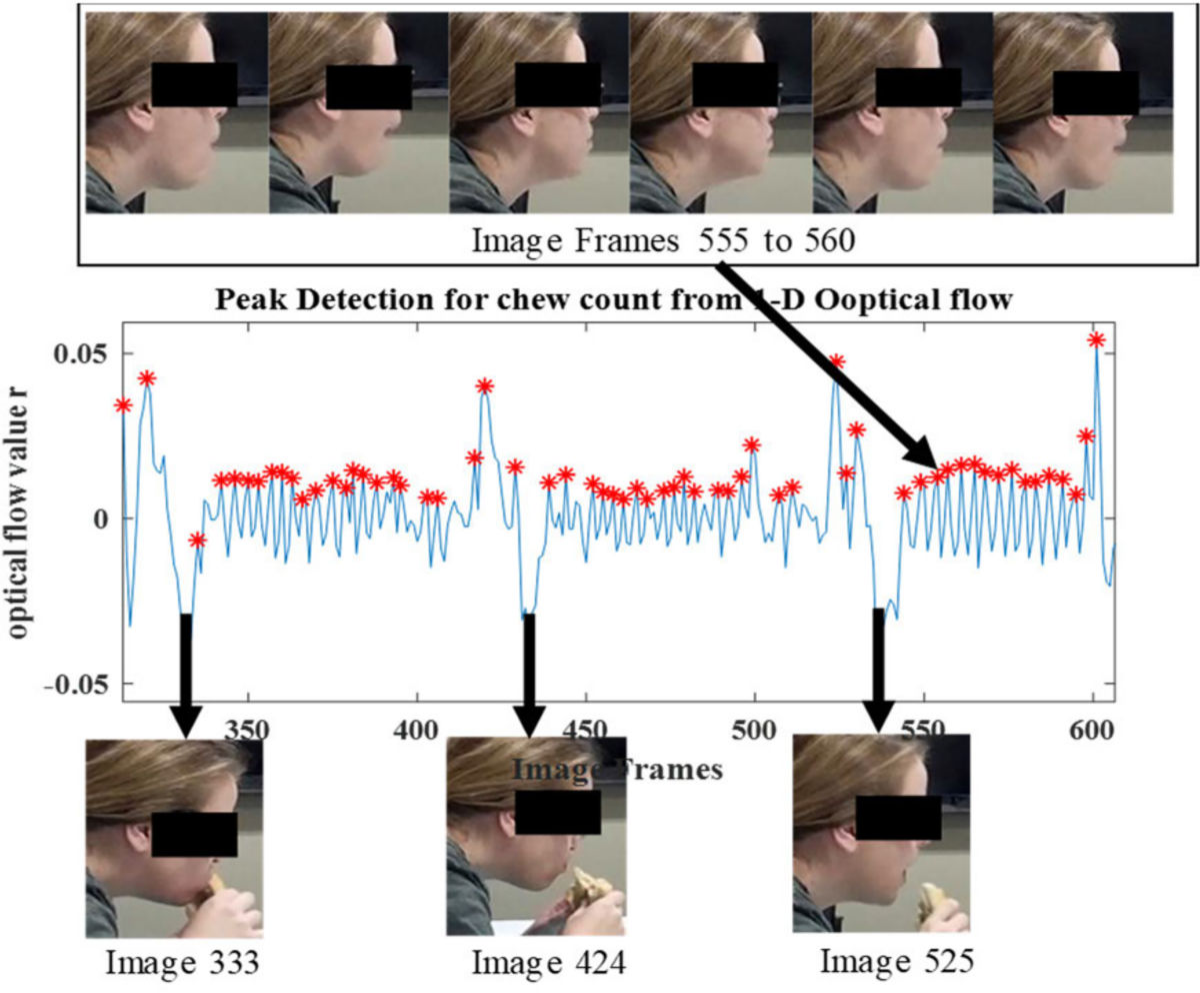
Effect of bite and chew in optical flow values.

**FIGURE 11.**
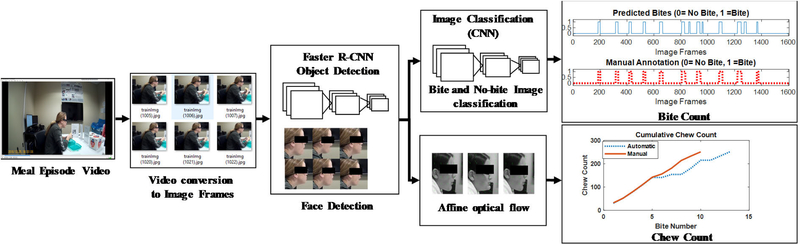
The pipeline of our developed method. First the input meal episode video is converted into 6 fps image frames. Then the image frames are passed through face detector. The detected faces is then classified into bite and no-bite images and consecutively affine optical flow algorithm is applied in detected faces to count number of chews within a bite.

**FIGURE 12.**
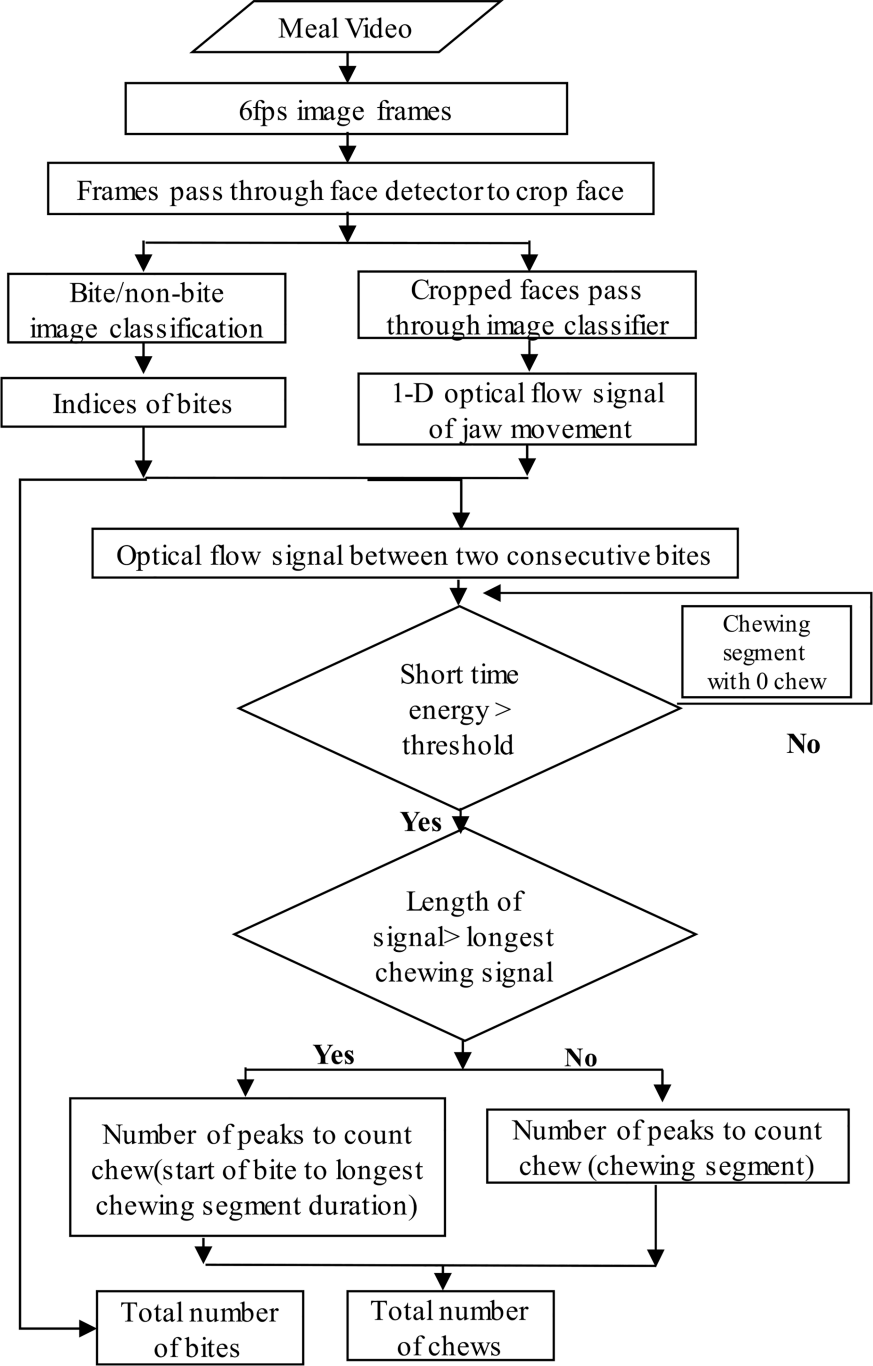
Flowchart of developed bite and chew counting algorithm from meal video.

The chew counts for all identified chewing segments were computed by finding peaks and counting them. A peak was defined as the highest point around which there are points lower by X (threshold = 0.5) on both sides. The number of peaks in the chewing segment represents the number of chews. Fig. 10 shows the optical flow parameter values corresponding to bite and chews. The figure shows a bite manifesting as a large spike in the value of optical flow, followed by a chewing sequence which produces oscillating values of optical flow.

## III. RESULTS

The performance evaluation of face detector utilized IoU (Intersection over Union) and mAP (mean Average Precision) metrics. IoU was calculated from the area of the overlap between the predicted bounding box and ground-truth bounding box, and the area encompassed by both the predicted bounding box and the ground-truth bounding box using the following formula.

$$IoU=\frac{Area\;of\;Overlap}{Area\;of\;Union}$$

mAP is the average AP (Average Precision) calculated for each class where AP is the area under the precision-recall curve.

$$AP=\int_{0}^{1}p(r)\;dr$$

$mAP=\frac{1}{N}\sum_{i=1}^{N}AP_{i}$ The trained face detector achieved an average IoU of 0.97±0.01 and an average mAP of 0.91±0.02 in 4-fold validation.

The cumulative confusion matrix for 4-fold cross-validation of image classifier to detect bite/non-bite images is shown in TABLE 1. The classifier achieved an average F1-score (± standard deviation) of 98.5% ± 1.5%, average precision of 98.5% ± 1.5%, sensitivity 99.1% ± 0.9% in training. Using the image classifier, we achieved an accuracy of 82.65%±8.70% in bite counts.

The chew counting algorithm achieved an accuracy of 88.64% ± 5.29% in 84 meal videos. Chews were counted by detecting peaks in the chewing segment of affine optical flow rotational values.

## IV. DISCUSSION

In this manuscript we proposed a method for automatic measurement of meal microstructure (bites and chews) from the video of eating episodes. The proposed method detects faces, identifies face images with a bite taking place, identifies chewing segments between consecutive bites and counts chews from affine optical flow parameters.

A bite is a phenomenon of placing the food (liquid/solid) in the mouth. During chewing the jaw makes grinding motion which can be inferred from the movement of pixels of the face in consecutive image frames. In finding both bite and chew in images, face is our region of interest (ROI).

To extract the ROI from image frames of meal videos, we applied Faster-RCNN object detection in each image frame. In order to reduce false detection, the threshold for confidence level was set as 0.80. The face detector was able to correctly identify the face in 385892 images among 388074 image frames. In cases, when participants moved away from the cameras field of view or blurriness in the images caused by the movement of participants then, face detector misses to detect the face.

The number of bites in the meal was identified from the detected faces using the trained image classifier. Using the image classifier, we achieved an accuracy of 85.4%±6.2% in counting bites for 84 meal videos of 28 subjects. The total number of predicted bites was 1903, whereas the total number of manually annotated bites was 1887 in the dataset. The mean number of bites in an eating episode from manual annotation was 22.5±10.3 (manual annotation) bites and 22.7±11.1 (image detection) bites.

Gestures which comprise bringing hand toward mouth such as the use of napkin to wipe the mouth during eating contribute in false prediction of bite count. Fig. 14(a) shows the false bite detection because of gestures mimicking the bite. Top graph shows the automatic bite count from image classification and bottom graph represents the manual annotation of bite/non-bite images in the eating episode.

**FIGURE 13.**
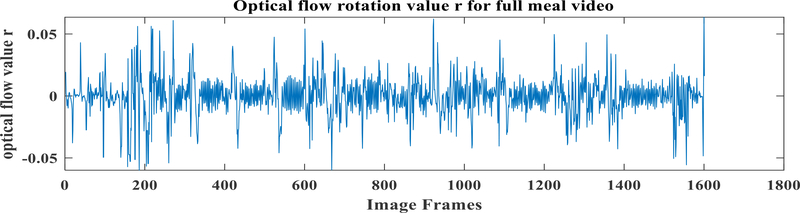
1-D optical flow values from cropped images of an eating episode.

The movement of the jaw during chewing can be inferred from the movement of pixels in the face in consecutive image frames. To find the movement of pixels in consecutive cropped faces, the affine optical flow was calculated and the rotational parameter of pixels’ movement was used to describe the chewing. Fig. 13 shows the calculated rotational parameter of affine optical flow in an eating episode.

To remove the effect of false prediction of bites from gestures mimicking a bite and drinking which are not followed by chewing, short-time energy of the optical flow signal between consecutive bites were calculated and thresholded to identify the chewing segment. Talking and movement of the head during these non-chewing segments often contribute enough in short time energy to cross the threshold and hence cause false chewing segment detection.

**FIGURE 14.**
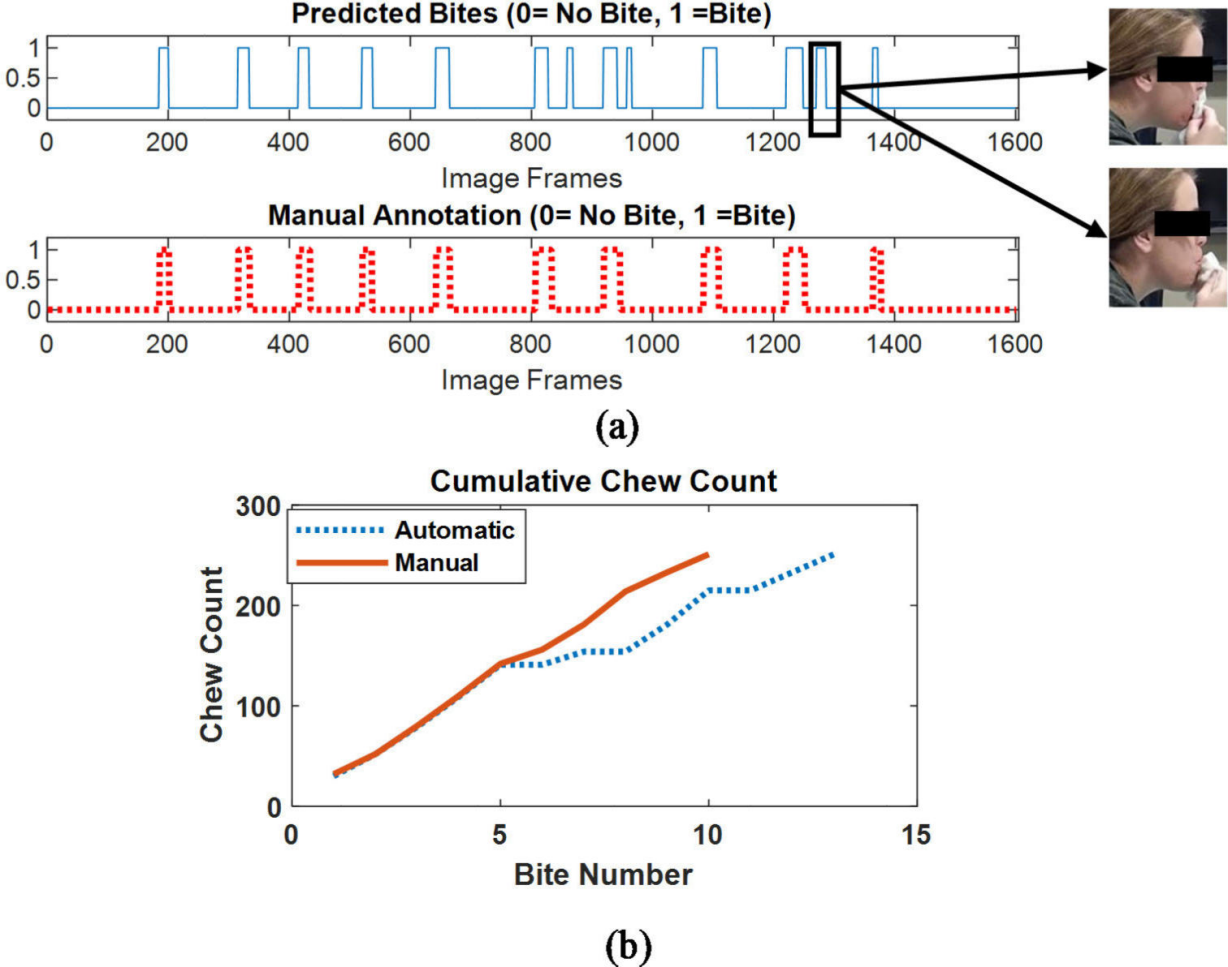
(a) False bite event detection because of gestures mimicking bite (b) Cumulative chew count from manual annotation and automatic count.

The developed chew counting algorithm achieved an accuracy of 88.9% ± 7.4% in counting the number of chews in 84 meal videos from 28 subjects. The total number of manually counted chew is 40057 and the total number of predicted chews from the proposed method is 41224. The average number of chews in an eating episode as per manual annotation is 476.9±228.7. The mean number of predicted chews by the automatic method in an eating episode is 490.8±248.4. False detection of bite and chewing segment because of gestures, contribute to overprediction of chew count. Also, several participants (up to 3) were invited to take a meal at a time and allowed to talk during eating, which contributed in false chewing detection and over counting of chews. Fig. 14(b) represents the cumulative chew count of an eating episode for both manual and automatic annotation.

The proposed method of counting bites and chews achieved an accuracy of 85.4%±6.2% in counting bites and 88.9% ± 7.4% in counting chews in 84 videos of eating episodes.

These results show promising encouragement in further enrichment of the proposed method to apply as an alternative of manual annotation. The manual annotation of meal microstructure such as bite and chews from video consume an immense amount of time of researchers in large feeding studies. In this study, videos were played 5X slower than the original speed while annotating bite and chews. An eating episode spanning 10 min and 6 seconds took around 50 min and 30 seconds to annotate whereas the proposed method took around 22 minutes and 55 minutes to convert the videos into image frames, pass the image frames through face detector, save the cropped faces, apply image classification to count the number of bites and optical flow to count the number of chews in MATLAB 2018b environment installed on a Windows 10 computer equipped with an Intel Core i7 9th Generation CPU with 16GB DDR4 RAM, and an NVIDIA GeForce GTX 1070 GPU with 8GB memory. Note that the time required in manual annotation is spent by human annotator but a single click on the mouse is all required by the human in the computer-based annotation.

Thus the proposed automatic method of bite and chew counting will save both effort, time and increase the reliability considerably than manual annotation of feeding study.

The video of eating episodes was recorded from the side of the participant to keep the movement of the jaw during chewing most visible. Future work should concentrate on developing a more robust algorithm that will be able to count a number of bites and chews in an eating episode from the video of any angle. In this study, the calculation of bites and chews are done offline. Future works may focus on implementing the process in real-time, providing meal microstructure information (number of bites, number of chews, duration of chewing segments) immediately after the meal.

These results show promising encouragement in further enrichment of the proposed method to apply as an alternative of manual annotation.

## V. CONCLUSION

We present a novel computer vision-based method of finding total number of bites and chews in an eating episode from the recorded video. To the best of our knowledge, the developed method is the first computer vision-based method which is applied to count both number of bites and chews in same eating episode. The contribution of this work is threefold. (I) Development of a face detector based on Faster-RCNN object detection scheme to detect faces (regions of interest, ROI) in recorded meal videos. (II) Development of simple image classifier based on pre-trained AlexNet, to classify the detected faces as ‘bite’ image and ‘non-bite’ image and hence calculating total number of bites in the eating episode. (III) To count number of chews in an eating episode by applying optical flow in consecutive cropped faces to find the movement of jaw. The developed method was applied on a very large dataset that contains 19 hours and 26 minutes of recorded meal video of 84 eating episodes from 28 subjects. A mean accuracy of 88.9% ± 7.4% in respect to manual annotation in counting the number of bites and a mean accuracy of 88.64 ± 5.29% for the number of chews in the 84 meal videos shows promising result which paves the way for further exploration to make the annotation of bites and chews in eating episodes fully automatic, accurate, less time consuiming and easy to use.

## Figures and Tables

**TABLE 1.** Cumulative confusion matrix of trained bite/non-bite image classifier.

		Predicted Class	
		No Bite	Bite
Actual Class	No Bite	5560	40
	Bite	40	2760

**TABLE 2.** Performace evaluation of face detection, bite count and chew count scheme.

Subject	No. of Image Frames	No. of detected face frames	No. of face detection accuracy (%)	Automatic Chew Count	Manual Chew Count	Accuracy (%)	Automatic Bite Count	Manual Bite Count	Accuracy (%)
**Sub01**	15014	14972	99.7	1500	1595	94.04	52	60	86.67
**Sub02**	15084	14972	99.3	1500	1594	94.10	52	56	92.86
**Sub03**	11474	11378	99.2	1056	1049	99.33	44	39	87.18
**Sub04**	14361	14291	99.5	1205	1153	95.49	67	60	88.33
**Sub05**	17770	17727	99.8	1324	1199	89.57	63	57	89.47
**Sub06**	15414	15369	99.7	1647	1313	74.56	70	65	92.31
**Sub07**	17972	17853	99.3	2040	1979	96.92	92	87	94.25
**Sub08**	18426	18341	99.5	2120	2056	96.89	108	106	98.11
**Sub09**	5810	5760	99.1	648	687	94.32	23	22	95.45
**Sub10**	9294	9229	99.3	777	713	91.02	49	53	92.45
**Subll**	13449	13359	99.3	1059	926	85.64	54	52	96.15
**Subl2**	7497	7482	99.8	572	538	93.68	35	31	87.10
**Sub13**	10028	9877	98.5	1003	935	92.73	54	52	96.15
**Subl4**	12472	12429	99.7	1545	1523	98.56	65	61	93.44
**Sub15**	10098	10009	99.1	1174	1220	96.23	52	52	100.00
**Subl6**	14380	14318	99.6	1781	1733	97.23	91	94	96.81
**Subl7**	179531	178526	99.4	20273	19844	97.84	932	940	99.15
**Subl8**	11523	11469	99.5	1517	1678	90.41	75	84	89.29
**Subl9**	15206	15097	99.3	2024	2126	95.20	108	121	89.26
**Sub20**	12663	12623	99.7	1932	1907	98.69	83	82	98.78
**Sub21**	30222	30105	99.6	2654	2317	85.46	113	100	87.00
**Sub22**	11548	11524	99.8	1616	1467	89.84	62	60	96.67
**Sub23**	19403	19329	99.6	1974	1748	87.07	94	81	83.95
**Sub24**	23578	23405	99.3	2033	2020	99.36	103	100	97.00
**Sub25**	8447	8404	99.5	839	895	93.74	45	50	90.00
**Sub26**	12682	12575	99.2	1713	1786	95.91	66	69	95.65
**Sub27**	12071	11978	99.2	1316	1385	95.02	59	58	98.28
**Sub28**	9670	9563	98.9	883	949	93.05	48	63	76.19
**Average**	**99.4**	**1472.3**	**1430.6**	**92.9**	**68.0**	**67.4**	**92.3**	**99.4**	**1472.3**
**Std**	**5024.9**	**5008.9**	**0.3**	**509.8**	**479.9**	**91.6**	**23.5**	**23.0**	**82.3**
